# [6]-Shogaol and [6]-Gingerol active ingredients may improve neuropathic pain by suppressing cytokine levels in an experimental model

**DOI:** 10.55730/1300-0144.5728

**Published:** 2023-10-31

**Authors:** Fikri ÖZDEMİR, Güven AKÇAY, Sevil ÖZKINALI, Çağla ÇELİK

**Affiliations:** 1 Department of Anatomy, Faculty of Medicine, Hitit University, Çorum, Turkiye; 2 Department of Biophysics, Faculty of Medicine, Hitit University, Çorum, Turkiye; 3 Department of Chemistry, Faculty of Arts and Sciences, Hitit University, Çorum, Turkiye; 4 Pharmacy Services Program, Vocational School of Health Services, Hitit University, Çorum, Turkiye

**Keywords:** [6]-shogaol, [6]-gingerol, ginger, neuroinflammation, neuropathic pain

## Abstract

**Background/aim:**

Neuropathic pain (NP) is a type of chronic pain usually caused by damage to the somatosensory system. Bioactive antioxidant compounds, such as curcumin and ginger, are widely preferred in the treatment of NP. However, the ingredient-based mechanism that underlies their pain-relieving activity remains unknown. The aim of this study was to investigate the therapeutic effects of trans-[6]-Shogaol and [6]-Gingerol active ingredients of the *Zingiber officinale* Roscoe extract on the spinal cord and cortex in the neuroinflammatory pathway in rats with experimental sciatic nerve injury.

**Materials and methods:**

Forty-six volatile phenolic components were identified in ginger samples using gas chromatography–mass spectrometry analysis. Thirty 3-month-old male 250–300 g Wistar Albino rats were divided into three groups as (i) sham, (ii) chronic constriction injury (CCI), and (iii) CCI+ginger. NP was induced using the CCI model. A ginger extract treatment enriched with trans-[6]-shogaol and [6]-gingerol active ingredients was administered by gavage at 200 mg/kg/day for 7 days. On the 14th day of the experiment, locomotor activity was evaluated in open field and hyperalgesia in tail flick tests.

**Results:**

In behavioural experiments, a significant decrease was observed in the CCI group compared to the sham group, while a significant increase was observed in the CCI+ginger group compared to the CCI group (p < 0.05). In the spinal cord and cortex tissues, there was a significant increase in the TNF-α, IL-1β, and IL-18 neuroinflammation results of the CCI group compared to the sham group, while there was a significant decrease in the CCI+ginger group compared to the CCI group.

**Conclusion:**

In this study, ginger treatment was shown to have a therapeutic effect on neuroinflammation against sciatic nerve damage.

## 1. Introduction

Neuropathic pain (NP) is a type of chronic pain that is usually caused by damage to the somatosensory system [1]. Approximately 20% of the adult population worldwide suffers from chronic pain each year [2], and neuroinflammation has an important role in the development of chronic pain. Neuroinflammation is also an underlying cause of many central nervous system (CNS) diseases, including Alzheimer’s disease, Parkinson’s disease, multiple sclerosis and psychiatric disorders [3]. In neurodegenerative diseases and spinal cord injury, neuroinflammation is a consequence of direct damage to the CNS, causing further neuronal degeneration and cell death (i.e. secondary injury) [3]. In chronic pain (i.e. neuropathic and inflammatory pain), neuroinflammation is usually due to peripheral damage and excessive neuronal activity of primary sensory neurons. Therefore, CNS neuroinflammation after peripheral injury is relatively mild and does not cause marked neuronal loss [4,5]. Cytokines such as tumour necrosis factor (TNF-α) and interleukin-1β (IL-1β) cause neurodegeneration in various regions of the brain associated with brain dysfunction in neurodegenerative disease (hippocampus and dentate gyrus) and impair memory and synaptic plasticity [6,7]. In contrast, TNF-α and IL-1β act as neuromodulators in the spinal cord dorsal horn after peripheral injury and trigger or enhance synaptic plasticity as well as inflammatory and NP [8–10].

Chronic pain, including NP caused by nerve injury and spinal cord injury, inflammatory pain caused by arthritis, cancer pain, and pain caused by drug therapy, are all caused by neuroinflammation in the spinal cord [11]. This neuroinflammation is triggered by activity-dependent release of glial activators (neurotransmitters, chemokines, and proteases) from the central terminals of primary afferent neurons and/or disruption of the blood–brain barrier [11]. Furthermore, neuroinflammation produces antiinflammatory cytokines and pro-resolution lipid mediators to normalise neuroinflammation, synaptic plasticity and abnormal chronic pain [11]. TNF-α is one of the most widely studied and potent inflammatory cytokines and has been shown to be expressed by microglia, astrocytes, and primary sensory dorsal root ganglion neurons [12,13]. IL-1β, another important inflammatory cytokine, is expressed by both microglia and astrocytes in the spinal cord [14,15], while IL-18 is induced in microglia after nerve injury and chronic opioid exposure [16,17]. TNF-α increases excitatory currents, IL-6 decreases inhibitory currents, and IL-1β increases excitatory currents and decreases inhibitory currents [18].

Among the different types of chronic pain, NP, caused by damage to the nervous system, including peripheral fibres and central neurons, is very difficult to treat and affects 7%–10% of the general population [19]. Current treatment options for NP are limited and opioid analgesics have serious side effects and can cause opioid use disorder. Recent studies have revealed the role of bioactive compounds in the diet in reducing NP. We evaluated the effects of commonly consumed bioactive compounds (ginger, curcumin, omega-3 polyunsaturated fatty acids, epigallocatechin gallate, and resveratrol) on NP and NP-related neuroinflammation [19]. Cellular studies have shown that these bioactive compounds reduce inflammation through the suppression of NF-κB and MAPK signalling pathways that regulate apoptosis/cell survival, antioxidant, and antiinflammatory responses. Animal studies strongly suggest that, when consumed regularly, these bioactive compounds have a pronounced antiNP effect as demonstrated by reduced mechanical allodynia, mechanical hyperalgesia, thermal hyperalgesia, and cold hyperalgesia [19].

Ginger (*Zingiber officinale* Roscoe) consists of a complex combination of biologically active components, among which the compounds ginger, shogaol, and paradol are reported to account for the majority of its antiinflammatory properties. Various ginger compounds and extracts have been tested as antiinflammatory agents, where the lengths of the side chains determine the level of efficacy [19]. β-[6]-gingerol, a combination of gingerols, is more effective than individual compounds in reducing inflammatory mediators. Borgonetti et al. showed that 200 mg/kg ginger once daily by gavage for 7 days starting from the 3rd day of nerve injury improved mechanical and thermal allodynia in mice with sciatic nerve injury [20]. In this study, the chronic constriction injury (CCI) model defined by Bennett and Xie [21] was used to induce NP and ginger treatment was started on the 7th day after surgery.

Chronic pain can be neuropathic or inflammatory. NP results from damage to the peripheral nervous system (PNS) or the CNS. Chronic NP burden has been shown to be associated with the complexity of NP symptoms, including anxiety and depression, and with poor outcomes [19]. Nerve injury often leads to neuroplastic changes in the peripheral and central elements of the pain system, resulting in neuronal hyperexcitability and sensitisation that produce spontaneous and evoked pain, such as mechanical and thermal hypersensitivity. The current symptomatic therapies for the treatment of NP rarely focus on the actual causes and have long-term side effects that limit treatment [20]. *Zingiber officinale* Roscoe (Zingiberaceae), known as ginger, is included in many official pharmacopoeias of different countries and contains nonvolatile components with biological activity, such as gingerols, shogaols, and paradols, along with zingerone in the dried rhizome [20]. Nerve damage in NP causes neuroinflammation and neuroplastic alterations in the peripheral and central neurons associated with sensitization and hyperexcitability [22]. It has been found that NP may result in an imbalance between reactive oxygen species (ROS) and endogenous antioxidants, which can cause neuroinflammation after nerve damage [22]. Therefore, there is an urgent need to develop new effective and safe analgesic and antiinflammatory alternatives without side effects [22].

The antiNP effect of bioactive compounds can be attributed to their ability to interact directly or indirectly with PNS and CNS signalling through their antiinflammatory and antioxidant properties. In this study, the therapeutic effects of ginger extract enriched in phenolic compounds, such as shogaol and gingerol, on neuroinflammation in the PNS and CNS were investigated in rats with experimental sciatic nerve injury.

## 2. Materials and methods

### 2.1. Animals and experimental design

**Figure 1:**
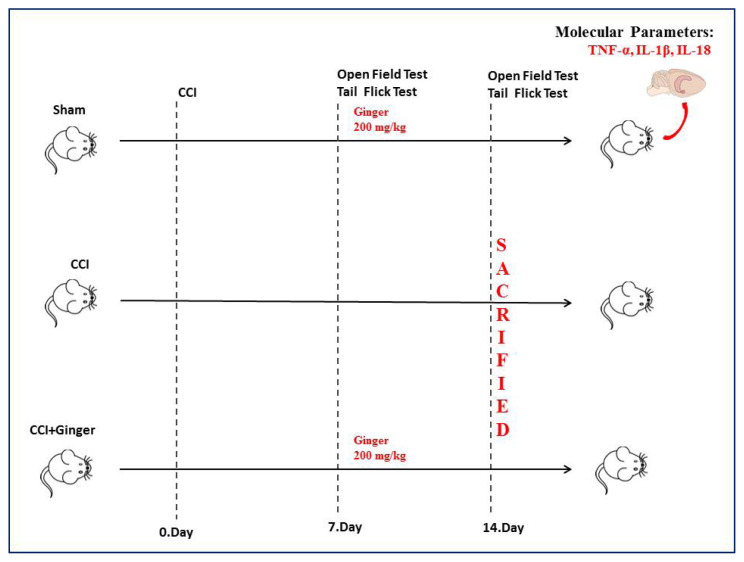
Experimental design.

Three-month-old male Wistar Albino rats weighing 250–300 g were used. Three experimental groups were formed as (i) sham, (ii) CCI, and (iii) CCI+ginger (Figure 1). NP was induced using the CCI model [21]. Animals in the sham and CCI+ginger groups were treated with ginger extract by gavage at a dose of 200 mg/kg/day for 7 days. Ginger 200 mg/kg, which is an effective dose in the treatment of sciatic nerve injury, was used in this study [20]. On 7th day and the 14th day of the experiment, locomotor activity was measured using an open field test and hyperalgesia was evaluated using the tail flick test. After the behavioural experiments, the rats were sacrificed and the levels of TNF-α, IL-1β, and IL-18 in the spinal cord and cortex tissues were evaluated using the ELISA method.

### 2.2. CCI surgery

The CCI model described by Bennett and Xie (1988) was used for the induction of NP [21]. In brief, rats were anaesthetized with isoflurane (5% for induction, 2.5% for maintenance), and a 1-cm incision was made along the longitudinal axis of the right hind leg distal to the hip, 3–4 mm below the femur. Then, 4 loose ligatures (4/0 chromic catgut) were tied proximal to the sciatic trifurcation approximately 1 mm apart. The ligations were loosened to minimize nerve constriction and allow epineural blood flow. After the procedure, the surgical incision was immediately sutured, and a povidone–iodine solution was applied externally. The rats were housed in separate cages for 4 h after the CCI surgery and were allowed to recover for 1 week before treatments. For the sham group, the sciatic nerve was exposed, similar to the CCI model, but no ligatures were placed. On the 14th day, the pain tests were completed and the cortex and spinal cord tissues of the subjects were taken.

### 2.3. Ginger extraction

The ginger powder was obtained from Bağdat Co. Ltd. in Türkiye and authenticated by Dr Sevil Özkınalı. A series of voucher specimens were stored at the Hitit University Department of Chemistry [23]. Extraction was carried out at 100 °C in a Soxlet device by adding 300 mL of ethyl alcohol to 4.0 g of powdered ginger [24,25]. Ethyl alcohol, a polar protic solvent, is preferred for the extraction of polar phenolic compounds such as shogaols and gingerols. At the end of the experiment, the ethyl alcohol is evaporated with the help of an evaporator and the remaining viscous mixture is weighed. A similar process is repeated with water and methyl alcohol to release the phenolic compounds in ginger.

### 2.4. Behavioural tests

#### 2.4.1 Open field test

Locomotor activity was carried out in a setup with a base of 80 × 80 cm and a wall height of 40 cm. For rats to explore the apparatus, they were placed in the centre of the field and monitored and recorded by the video surveillance system (Noldus Ethovision XT System, Netherlands) for 5 min. The total distance (cm) and frequency of movement were calculated to evaluate locomotor activity [26].

#### 2.4.2 Tail flick test

Thermal hyperalgesia was also evaluated by the tail flick test, in which the animal’s tail is exposed to a heat source [27]. When the animal feels uncomfortable, it automatically raises its tail. Briefly, the 2 cm portion of the distal tail was immersed into a 52.5 ± 0.2°C water bath. The time the rats took to flick their tail was recorded as tail flick latency; the cutoff latency was 15 s to avoid injury of the tissues of the tail [28].

### 2.5. Tissue collection

The animals were killed by decapitation on day 14 after the behavioural experiments. The whole cerebral cortex and ipsilateral or contralateral spinal cord (T7/8-L5) were dissected from the brain and stored frozen at −80 °C. The tissues were homogenized in phosphate buffered saline (pH 7.4), centrifuged at 12,000 rpm for 20 min at 4 °C, and the supernatants were used for biochemical analyses.

### 2.6. Biochemical analysis

#### 2.6.1. Enzyme-linked immunosorbent assay (ELISA)

The levels of TNFα, IL-1β, and IL-18 were quantified using commercially available ELISA kits (R&D Systems, MN, USA) for rat TNF-α (EK710127), IL-1β (EK710260), and IL-18 (EK710281) according to the manufacturer’s instructions. The concentrations of TNF-α, IL-1β, and IL-18 in the samples were calculated from their corresponding absorbance values via the standard curve. The data were normalized to total tissue protein and expressed as pg mg^−1^ tissue protein.

#### 2.6.2 Protein measurements

Protein concentrations were measured in the sample tissues at 595 nm by a modified Bradford assay using a Coomassie Plus reagent with a bovine serum albumin standard (Pierce Chemical Company, Rockford, IL, USA).

#### 2.6.3 Gas chromatography–mass spectrometry (GC–MS) analysis

**Figure 2:**
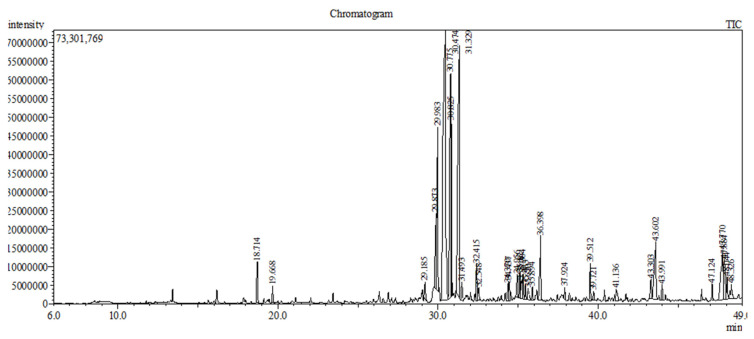
The total ion chromatograms of ginger extract as determined via GC–MS.

A Shimadzu GCMS QP 2010 ULTRA with a high-performance quadrupole mass filter was used for the detection of volatile phenolic compounds in *Zingiber officinale*. An RXI-5MS capillary column (30 m; 0.25 mm; 0.25 *μ*m) was used. For the GC–MS analysis, the temperature of the injection port was set to 250 °C, the split ratio was adjusted to 25:1, and the carrier gas was helium (99.999 % pure) with a flow rate of 1.0 mL/min. The oven temperature program started at 40 °C for 3 min, and was then raised to 240 °C at a rate of 4°C/min, where it remained for 10 min. The analysis was completed in 63 min. The working solutions of *Zingiber officinale* were prepared at 1 mg/mL in ethanol. For the MS (Shimadzu with a high-performance quadrupole mass filter) analysis, the ion source temperature was set to 200 °C, and the transfer line temperature was set to 250 °C (Figure 2).

#### 2.6.4 Qualitative and quantitative analysis of Zingiber officinale by GC–MS

We detected 46 volatile phenolic compounds in the ginger samples (Bağdat Co. Ltd., Türkiye). The total ion chromatograms and detailed information on various compounds are shown in the Table. In the GC–MS analysis, a broad array of masses was obtained within a scan time of 5.0 to 60.3 minutes (scan range 20–450 m/z) and the presence of metabolites was determined with various retention times. The types of metabolites were verified by comparing the generated spectral model with those of the built-in spectral library developed by the National Institute of Standards and Technology (NIST, Washington, DC, USA), data version W9N11. As seen in the Table, most of the identified metabolites belong to the 5 main classes of sesquiterpenes (38.26%), phenolic compounds (29.95%), terpenes (8.08%) and their derivatives (14.3%), and fatty acids (6.4%). The superior performance of ginger in the aforementioned antioxidant assay can be attributed to the presence of several metabolites in the sesquiterpene, phenolic, and terpenoid classes, as profiled in the Table. The main aroma components of ginger are 11 phenolics and 7 sesquiterpenes, and it has been determined that there are 13 terpenes, 4 fatty acids, 4 terpenoids, 5 sesquiterpene alcohols, 1 aldehyde, and 1 alcohol derivative, among other components.

### 2.7. Statistical analysis

SPSS version 20.0 was used for all analyses. The results are given as mean ± standard error of the mean (SEM), and p values less than 0.05 were considered significant. One-way analysis of variance (ANOVA) was used for the data analysis with normality conditions checked using the Shapiro–Wilk test. The Tukey test was used for posthoc analysis.

## 3. Results

### 3.1. Effects of ginger on locomotor activity and thermal hyperalgesia

**Figure 3:**
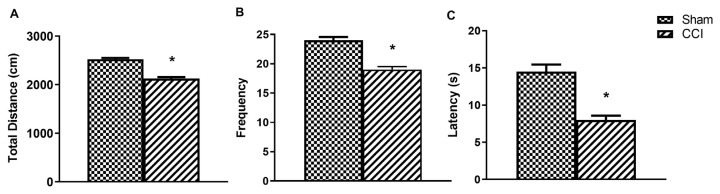
Day 7 baseline open field (OF) test and tail flick (TF) test results. (a) Total distance (cm) from OF, (b) frequency in OF, (c) latency (s) in TF. The * indicates significant difference (p < 0.05) compared to the sham group. All data are presented as mean ± SEM.

**Figure 4:**
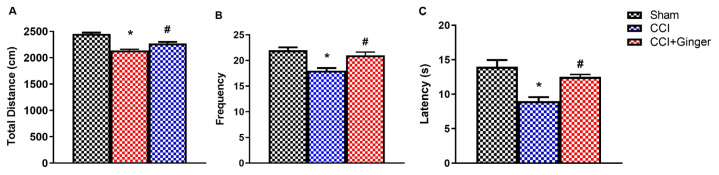
Day 14 baseline OF test and TF test results (n = 10 for each group). (a) Total distance (cm) from OF, (b) frequency in OF, (c) latency (s) in TF. The * indicates a significant difference (p < 0.05) from the sham group, and the # indicates a significant difference (p < 0.05) from the CCI group, based on one-way ANOVA followed by a Tukey posthoc test. All data are presented as mean ± SEM.

The total distance (cm) and frequency values, the determinants of the locomotor scores from the open field test, were significantly decreased in the CCI group on the 7th day compared to the sham group (p < 0.05) (Figures 3a and 3b). Similarly, the tail flick latency of the CCI rats was significantly reduced in the tail flick test on the 7th day, as compared to the sham group (p < 0.05) (Figure 3c). On the 14th day, a significant decrease was observed in the total distance (p < 0.05), frequency (p < 0.05), and latency (p < 0.05) values of the CCI group compared to the sham group, while a significant increase was observed in the total distance, frequency and latency values of the CCI+ginger group compared to the CCI group (Figure 4).

### 3.2. TNFα, IL-1β, IL-6 and IL-18 levels in cerebral cortex and spinal cord tissues

**Figure 5:**
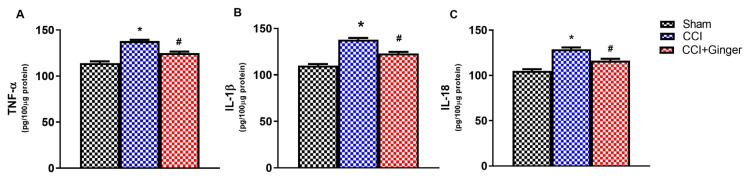
Neuroinflammation results in the cerebral cortex for (a) TNF-α levels, (b) IL-1β levels, and (c) IL-18 levels (n=10, for each group). The * indicates a significant difference (p < 0.05) from the sham group, and the # indicates a significant difference (p < 0.05) compared to the CCI group, based on one-way ANOVA followed by a Tukey posthoc test. All data are presented as mean ± SEM.

**Figure 6:**
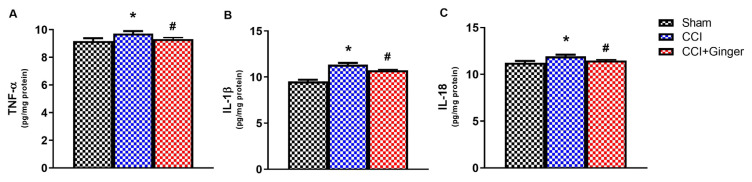
Neuroinflammation results in spinal cord. A) TNF-α levels, B) IL-1β levels, C) IL-18 levels. (n=10, for each group; * p<0.05, shows the difference compared to the Sham group, # p<0.05 shows the difference compared to the CCI group, one-way ANOVA test, followed by Tukey post hoc test). All data are presented as means ± SEM.

The levels of TNF-α (p < 0.05), IL-1β (p < 0.05), and IL-18 (p < 0.05) in the cerebral cortex and spinal cord are shown in Figures 5 and 6. The levels of TNF-α, IL-1β, and IL-18 in the cerebral cortex of the CCI group were significantly increased compared to the sham group (p < 0.05). The ginger treatment significantly decreased all the proinflammatory cytokine levels in the cerebral cortex tissues of the CCI rats on the 14th day (p < 0.05 for all). The levels of TNF-α (p < 0.05), IL-1β (p < 0.05), and IL-18 (p < 0.05) in the spinal cord of the CCI group were also significantly increased compared to the sham group. The ginger treatment significantly decreased all the proinflammatory cytokine levels in the spinal cord tissues of the CCI on the 14th day (p < 0.05 for all).

**Figure 7:**
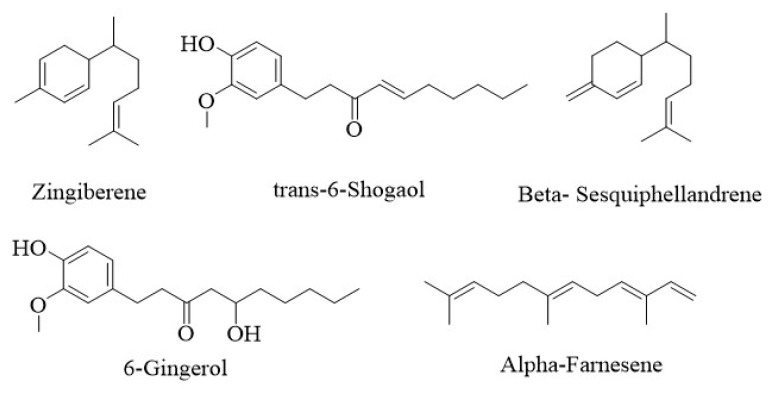
The 5 main components of ginger extract.

Zingiberene (18.18%), trans-[6]-shogaol (13.34%), β-sesquiphellandrene (10.26%), [6]-gingerol (6.37%), and (E,E)-α-farnesene (6.05%) compounds (Figure 7) were determined by the GC–MS analyses to be the most abundant compounds in the ginger extract. Among these main components, zingiberene, β-sesquiphellandrene and (E,E)-α-farnesene are in the sesquiterpene family, while trans-[6]-shogaol and [6]-gingerol are phenolic compounds. In the literature, terpene derivatives have been found to reduce neuroinflammation through HDAC-1 inhibition in a mouse neuropathy model [29]. Also, zingiberene, a sesquiterpene derivative, was found to be the most promising HDAC1 inhibitor [29]. Zingiberene has various pharmacological properties such as anticancer [30], antioxidant [31], antiulcer [32], antiviral [33], and antibacterial [34] effects. Among the other main components, phenolic compounds such as shogaol, gingerol, gingerdione, 2-cuparenol, carinol, nortrachelogenin, and zingerone were detected in the ginger extract. These compounds are known to have antioxidant and antiinflammatory activities [35]. Terpenoids, like phenolic compounds, have been extensively studied in the literature, and the evidence of their antioxidant potential is well recorded [36].

## 4. Discussion

NP is chronic pain caused by somatosensory damage. Approximately 20% of the adult population worldwide suffers from chronic pain each year. ROS generation and inflammation play an important role in the NP mechanism. Although anticonvulsants, antidepressants, opioids, and nonopioids are used in the treatment of NP, there is no drug with proven efficacy. Antioxidant bioactive compounds, such as curcumin and ginger, reduce inhibitory system activation and are used in pain treatment. These antioxidant compounds are frequently used as therapeutic agents affecting antiinflammatory pathways. The side effects of long-term use of current symptomatic therapies for the treatment of NP limit treatment and rarely focus on the actual causes [20]. *Zingiber officinale* Roscoe (Zingiberaceae), known as ginger, is included in many official pharmacopoeias of different countries and contains nonvolatile components with biological activity, such as gingerols, shogaols, paradols, and zingerone in the dried rhizome [20]. Ginger is a promising bioactive compound used in the treatment of NP due to its antiinflammatory properties [22]. As expected, the GC–MS analysis identified [6]-gingerol and [6]-shogaol as major components, as well as zingiberene, β-sesquiphellandrene, and (E,E)-α-farnesene [37]. Gingerols are the main pungent compounds found in the rhizomes of ginger (*Zingiber officinale* Roscoe), and gingerol analogues are thermally unstable and readily undergo dehydration reactions to form the corresponding shogaols, which give dried ginger its characteristic pungent taste. Both gingerols and shogaols exhibit a range of biological activities ranging from anticancer, antioxidant, antimicrobial, antiinflammatory and antiallergic to various CNS activities [37]. Gingerols and shogaols have been thoroughly studied for their antiinflammatory properties, especially concerning the reduction of NF-κBp65 activation and proinflammatory cytokines released from glial cells [20]. The injection of 10 μg [6]-gingerol into the rat spinal cord was found to be effective in relieving NP. [6]-Gingerol was also found to block prion peptide-mediated neurotoxicity associated with hypoxia-inducible factor 1a, while preserving mitochondrial function [37]. Specific primary sensory neurons include a functional vanilloid receptor accountable for the transmission of a pain or itch stimulus to the CNS. This receptor is activated by vanilloids such as capsaicin and high temperatures. It has been determined that [6]-gingerol inhibits capsaicin-induced contraction at a certain dose [37]. Excessive oxidative stress has been linked to the advance of chronic NP [22]. It has been demonstrated that both gingerols and shogaols significantly decreased ccf-mtDNA levels in NP animals treated with spinal nerve ligation [22]. In addition, it has been shown that ginger root extract increased antioxidant capacity and improved mitochondrial function by reducing ROS production in rats [22].

The relationship between ginger, which is known to have a therapeutic effect on locomotor activity and thermal hyperalgesia after NP, and neuroinflammation has not been fully elucidated. In the present study, the therapeutic effects of ginger on the spinal cord and cortex in the neuroinflammatory pathway were investigated in rats with experimental sciatic nerve injury.

We found that ginger treatment could alleviate pain behaviours in CCI rats by reducing proinflammatory cytokine production in cortex and spinal cord tissues. The experimental NP was induced using the CCI model, which is an easily reproducible and reliable method. CCI mimics traumatic mechanical injury in humans very well and demonstrates many of the pathophysiological features of chronic NP [38]. After the CCI surgery, the rats showed abnormal posture and licking of injured hindlimbs, resembling clinical neuropathic symptoms resulting from nerve injury in chronic pain patients [39]. Pain behaviours peaked on the 7th day after CCI [39], and distinctly nociceptive behaviours were apparent until day 14. [40]. Therefore, we evaluated the nociceptive pain behaviours on the 7th and 14th days after CCI surgery. We determined that locomotor activity (open field test) and thermal hyperalgesia (tail flick test) were affected at day 7 in the CCI rats. These results are consistent with previous studies demonstrating increased nociceptive behaviours due to sciatic nerve injury in rats following CCI [39,41,42]. We also determined that 200 mg/kg of ginger treatment provided improvements in the nociceptive behaviours of CCI rats. Borgonetti et al. reported in their study on mice with sciatic nerve damage that 200 mg/kg ginger treatment for 7 days, starting from the 3rd day of nerve damage, improved mechanical and thermal allodynia but did not change locomotor activity [20]. In addition, the characteristic of persistent NP mode is decreased locomotor activity [43]. In contrast, we observed that 200 mg/kg ginger treatment improved both locomotor activity and thermal allodynia. NP begins to occur on the 7th day after surgery in animals with nerve damage [2]. Borgonetti et al. started treatment on day 3 after surgery, whereas we started the ginger treatment on the 7th day after surgery and applied it for 7 days; this might explain the different results for improvement in locomotor activity.

Proinflammatory cytokines are important in the development and maintenance of NP [44]. NP is characterised by glial cell activation and proinflammatory cytokine secretion in the spinal dorsal horn [2]. TNF-α is one of the most potent proinflammatory cytokines expressed by microglia, astrocytes, and primary sensory dorsal root ganglion neurons [45]. IL-1β is another important inflammatory cytokine expressed by both microglia and astrocytes in the spinal cord [45]. Experimental studies have shown that TNF-α and IL-1β induce NP and that anticytokine therapy may be promising in the treatment of NP. It is also known that inflammatory changes in macrophages lead to the secretion of IL-18 as well as IL-1β in both the CNS and the PNS following CCI [46,47]. Cheng et al. reported that CCI injury increased IL-1β and IL-18 in the spinal cord and also decreased claw withdrawal latency and claw withdrawal threshold [39]. They also showed that treatment with loganin, an iridoid glycoside, decreased IL-1β and IL-18 in the spinal cord [39]. Likewise, Wen et al. found that CCI injury significantly decreased paw withdrawal latency and paw withdrawal threshold on days 7 and 14, while TNF-α, IL-1β, and IL-6 levels in the spinal cord were significantly increased [42]. Borgonetti et al. reported that 200 mg/kg ginger treatment inhibited NF-κB signalling activation and reduced the release of IL-1β, TNF-α, and IL-6 in CCI rats [20]. Consistent with these reports, our study showed that CCI injury significantly increased levels of TNF-α, IL-1β, and IL-18 in the cerebral cortex and spinal cord. Treatment with 200 mg/kg ginger for 7 days decreased the levels of TNF-α, IL-1β, IL-6, and IL-18 in the cerebral cortex and spinal cord tissue of CCI rats. Our results show that the ginger treatment regulates locomotor activity and thermal hyperalgesia in CCI rats by its antiinflammatory effects in the cerebral cortex and spinal cord.

Our study has some limitations. We only used male rats, not females. It is known that sex differences are important contributors to pain sensitivity and the analgesic efficacy of treatments. Another important limitation is that the long-term effects of phenolic compounds such as [6]-shogaol and [6]-gingerol obtained from ginger extract have not been investigated; understanding these is important for the application of treatments in clinical settings. In addition, investigating the free radical levels and apoptosis pathways that lead to neuroinflammation would have made a great contribution.

## 5. Conclusion

As a result of GC–MS quantitative analysis, trans-[6]-shogaol (13.34%) and [6]-gingerol (6.37%) were found to be the main phenolic components in ginger. This supports previous research that found the same results for [6]-gingerol and [6]-shogaol as a result of GC–MS analysis [37]. These components have been investigated for their antiinflammatory properties [20], and our study concurred, concluding that trans-[6]-shogaol and [6]-gingerol, as active ingredients in total ginger extract, may provide a treatment effect on weakened nociceptive behaviour and reduced thermal hyperalgesia caused by sciatic nerve damage. The findings showed that 200 mg/kg ginger extract treatment attenuated nociceptive behaviour and reduced thermal hyperalgesia caused by sciatic nerve injury. Ginger extract treatment, which is rich in phenolic components such as shogaols and gingerols, showed a therapeutic effect on NP by regulating cytokine levels. There is a need for antioxidant treatment strategies to be used alone or in combination with other effective therapies to alleviate NP in the future.

## Figures and Tables

**Table:** Identified components of the ginger extract

No	Compound	Formula	Retention time (min)	% area	Retention index	Classes
1	1,3-butanendiol	C_4_H_10_O_2_	5.192	0.68	0	alcohols
2	endo-borneol	C_10_H_18_O	18.714	0.87	1138	terpenes
3	α-terpineol	C_10_H_18_O	19.668	0.33	1143	terpenes
4	(−)-β-chamigrene	C_15_H_24_	29.185	0.37	1507	sesquiterpenes
5	R-α-curcumene	C_15_H_22_	29.873	2.05	1480	aromatic monoterpenoids
6	S-α-curcumene	C_15_H_22_	29.983	4.22	1524	aromatic monoterpenoids
7	zingiberene	C_15_H_24_	30.474	18.18	1496	monocyclic sesquiterpene
8	(E,E)-α-farnesene	C_15_H_24_	30.775	6.05	1458	sesquiterpenes
9	β-bisabolene	C_15_H_24_	30.825	2.40	1500	sesquiterpenes
10	β-sesquiphellandrene	C_15_H_24_	31.329	10.26	1523	sesquiterpenes
11	α-patchoulene	C_15_H_24_	31.493	0.41	1459	triterpene
12	*trans*-nerolidol	C_15_H_26_O	32.415	0.74	1564	sesquiterpenes
13	dodecanoic acid	C_12_H_24_O_2_	32.548	0.41	1570	saturated fatty acids
14	guaiol	C_15_H_26_O	34.370	0.43	1614	sesquiterpenoid alcohols
15	caryophyllene oxide	C_15_H_24_O	34.437	0.58	1507	terpenes
16	zingerone	C_11_H_14_O_3_	34.956	0.49	1638	phenolics
17	β-eudesmol	C_15_H_26_O	35.121	0.57	1656	terpenes
18	α-eudesmol	C_15_H_26_O	35.210	0.49	1598	terpenes
19	α-bisabolol	C_15_H_26_O	35.304	0.59	1688	sesquiterpene alcohols
20	(R,R)-α-bisabolol	C_15_H_26_O	35.463	0.27	1625	sesquiterpene alcohols
21	β-bisabolol	C_15_H_26_O	35.620	0.40	1619	sesquiterpene alcohols
22	bicyclo[4.3.0] nonane, 2,2,6,7- tetramethyl-7-hydroxy-	C_13_H_24_O	35.894	0.26	0	sesquiterpenoids
23	*E*-nerolidol	C_15_H_24_O	36.398	1.56	1572	sesquiterpene alcohols
24	2-cuparenol	C_15_H_22_O	37.924	0.24	1776	phenolics
25	campherenone	C_15_H_24_O	39.512	0.79	0	terpenes
26	2-methyl-5-(2,6,6-trimethyl-cyclohex-1-enyl)-pentane-2,3-diol	C_15_H_24_O	39.721	0.26	1776	terpenes
27	campherenone	C_15_H_24_O	41.136	0.46	0	terpenes
28	geranyl-*p*-cymene	C_20_H_30_	43.303	0.42	2006	terpenes
29	*n*-hexadecanoic acid	C_16_H_32_O_2_	43.602	2.61	1968	saturated fatty acids
30	geranyl-α-terpinene	C_20_H_32_	43.991	0.32	1962	terpenes
31	(−)-nortrachelogenin	C_20_H_22_O_7_	47.124	0.35	1328	phenolics
32	9,12-octadecadienoic acid (Z,Z)-	C_18_H_32_O_2_	47.770	2.72	2183	fatty acids
33	7-tetradecenal, (Z)-	C_14_H_26_O	47.884	1.50	1609	fatty aldehydes
34	geranyllinalool	C_20_H_34_O	48.044	0.59	2046	terpene alcohols
35	octadecanoic acid	C_18_H_36_O_2_	48.326	0.61	2167	fatty acids
36	(4-methoxy-phenyl)-(2-nitrocyclohexyl)-methanol	C_14_H_19_NO_4_	49.701	1.63	2148	aromatic terpenes
37	zingerone	C_11_H_14_O_3_	49.896	1.01	1638	phenolics
38	*trans*-6-Shogaol	C_17_H_24_O_3_	51.468	13.34	0	phenolics
39	ZO-3-(6)-gingerdione	C_17_H_24_O_4_	52.121	1.03	0	phenolics
40	butanoic acid, 3,7-dimethyl-2,6-octadienyl ester, (E)-	C_14_H_24_O_2_	52.350	0.33	1550	terpenes
41	6-gingerol	C_17_H_26_O_4_	53.518	6.37	2396	phenolics
42	carinol	C_20_H_26_O_6_	55.599	0.79	3296	phenolics
43	*cis*-8-shogaol	C_19_H_28_O_3_	56.163	4.43	0	phenolics
44	(E)-4-(2’,6’,6’-trimethyl-1’,2’-epoxy-cyclohexyl)-3-penten-2-one	C_14_H_22_O_2_	57.175	0.62	0	terpenes
45	gingerol	C_17_H_26_O_4_	59.208	0.68	2396	phenolics
46	1-(2,4-dihydroxyphenyl)-2-(4-methoxy-3-nitrophenyl)ethanone	C_15_H_13_NO_6_	60.299	1.22	2728	phenolics

## References

[1] Ngernyam N, Jensen MP, Auvichayapat N, Punjaruk W, Auvichayapat P (2013). Transcranial direct current stimulation in neuropathic pain. Journal of Pain & Relief.

[2] Akcay G, Nemutlu Samur D, Derin N (2023). Transcranial direct current stimulation alleviates nociceptive behaviour in male rats with neuropathic pain by regulating oxidative stress and reducing neuroinflammation. Journal of Neuroscience Research.

[3] Rivest S (2009). Regulation of innate immune responses in the brain. Nature Reviews Immunology.

[4] Polgár E, Gray S, Riddell JS, Todd AJ (2004). Lack of evidence for significant neuronal loss in laminae I-III of the spinal dorsal horn of the rat in the chronic constriction injury model. Pain.

[5] Scholz J, Broom DC, Youn DH, Mills CD, Kohno T (2005). Blocking caspase activity prevents transsynaptic neuronal apoptosis and the loss of inhibition in lamina II of the dorsal horn after peripheral nerve injury. Journal of Neuroscience.

[6] Vereker E, O’Donnell E, Lynch MA (2000). The inhibitory effect of interleukin-1beta on long-term potentiation is coupled with increased activity of stress-activated protein kinases. Journal of Neuroscience.

[7] Ren WJ, Liu Y, Zhou LJ, Li W, Zhong Y (2011). Peripheral nerve injury leads to working memory deficits and dysfunction of the hippocampus by upregulation of TNF-α in rodents. Neuropsychopharmacology.

[8] Liu YL, Zhou LJ, Hu NW, Xu JT, Wu CY (2007). Tumor necrosis factor-alpha induces long-term potentiation of C-fiber evoked field potentials in spinal dorsal horn in rats with nerve injury: The role of NF-kappa B, JNK and p38 MAPK. Neuropharmacology.

[9] Choi JI, Svensson CI, Koehrn FJ, Bhuskute A, Sorkin LS (2010). Peripheral inflammation induces tumor necrosis factor dependent AMPA receptor trafficking and Akt phosphorylation in spinal cord in addition to pain behavior. Pain.

[10] Park CK, Lü N, Xu ZZ, Liu T, Serhan CN (2011). Resolving TRPV1- and TNF-α-mediated spinal cord synaptic plasticity and inflammatory pain with neuroprotectin D1. Journal of Neuroscience.

[11] Ji RR, Xu ZZ, Gao YJ (2014). Emerging targets in neuroinflammation-driven chronic pain. Nature Reviews Drug Discovery.

[12] Schäfers M, Geis C, Svensson CI, Luo ZD, Sommer C (2003). Selective increase of tumour necrosis factor-alpha in injured and spared myelinated primary afferents after chronic constrictive injury of rat sciatic nerve. European Journal of Neuroscience.

[13] Xu JT, Xin WJ, Zang Y, Wu CY, Liu XG (2006). The role of tumor necrosis factor-alpha in the neuropathic pain induced by lumbar 5 ventral root transection in rat. Pain.

[14] Zhang RX, Liu B, Wang L, Ren K, Qiao JT (2005). Spinal glial activation in a new rat model of bone cancer pain produced by prostate cancer cell inoculation of the tibia. Pain.

[15] Clark AK, Staniland AA, Marchand F, Kaan TK, McMahon SB (2010). P2X7-dependent release of interleukin-1beta and nociception in the spinal cord following lipopolysaccharide. Journal of Neuroscience.

[16] Miyoshi K, Obata K, Kondo T, Okamura H, Noguchi K (2008). Interleukin-18-mediated microglia/astrocyte interaction in the spinal cord enhances neuropathic pain processing after nerve injury. Journal of Neuroscience.

[17] Cen ML, Cao H, Chu YX, Cheng LZ, Liang LL (2012). Role of P2X7 receptor-mediated IL-18/IL-18R signaling in morphine tolerance: multiple glial-neuronal dialogues in the rat spinal cord. The Journal of Pain.

[18] Ellis A, Bennett DL (2013). Neuroinflammation and the generation of neuropathic pain. British Journal of Anaesthesia.

[19] Shen CL, Castro L, Fang CY, Castro M, Sherali S (2022). Bioactive compounds for neuropathic pain: An update on preclinical studies and future perspectives. Journal of Nutritional Biochemistry.

[20] Borgonetti V, Governa P, Biagi M, Pellati F, Galeotti N (2020). *Zingiber officinale* Roscoe rhizome extract alleviates neuropathic pain by inhibiting neuroinflammation in mice. Phytomedicine.

[21] Bennett GJ, Xie YK (1988). A peripheral mononeuropathy in rat that produces disorders of pain sensation like those seen in man. Pain.

[22] Shen CL, Wang R, Ji G, Elmassry MM, Zabet-Moghaddam M (2022). Dietary supplementation of gingerols- and shogaols-enriched ginger root extract attenuate pain-associated behaviours while modulating gut microbiota and metabolites in rats with spinal nerve ligation. Journal of Nutritional Biochemistry.

[23] Tang W, Zhu SC, Tan XJ, Cao J, Ye LH (2023). Chemometrics and antioxidant activity assisted nontargeted metabolomics for the identification of ginger species. Journal of Pharmaceutical and Biomedical Analysis.

[24] Dalsasso RR, Valencia GA, Monteiro AR (2022). Impact of drying and extractions processes on the recovery of gingerols and shogaols, the main bioactive compounds of ginger. Food Research International.

[25] Tanweer S, Mehmood T, Zainab S, Ahmad Z, Shehzad A (2020). Comparison and HPLC quantification of antioxidant profiling of ginger rhizome, leaves and flower extracts. Clinical Phytoscience.

[26] Akçay G, Aslan M, Kipmen Korgun D, Çeker T, Akan E (2023). Effects of transcranial direct current stimulation on the glutamatergic pathway in the male rat hippocampus after experimental focal cerebral ischemia. Journal of Neuroscience Research.

[27] Hacısüleyman L, Saraç B, Joha Z (2022). Analgesic effects of vilazodone, indatraline, and talsupram in a rat model of neuropathic pain. Turkish Journal of Pharmaceutical Sciences.

[28] Arslan R, Aydin S, Nemutlu Samur D, Bektas N (2018). The possible mechanisms of protocatechuic acid-induced central analgesia. Saudi Pharmaceutical Journal.

[29] Borgonetti V, Governa P, Manetti F, Galeotti N (2023). Zingiberene, a non-zinc-binding class I HDAC inhibitor: A novel strategy for the management of neuropathic pain. Phytomedicine.

[30] Li J, Thangaiyan R, Govindasamy K, Wei J (2021). Anti-inflammatory and anti-apoptotic effect of zingiberene on isoproterenol-induced myocardial infarction in experimental animals. Human & Experimental Toxicology.

[31] El-Ghorab AH, Nauman M, Anjum FM, Hussain S, Nadeem M (2010). A comparative study on chemical composition and antioxidant activity of ginger (*Zingiber officinale*) and cumin (*Cuminum cyminum*). Journal of Agricultural and Food Chemistry.

[32] Singh PK, Kaur IP (2012). Synbiotic (probiotic and ginger extract) loaded floating beads: A novel therapeutic option in an experimental paradigm of gastric ulcer. Journal of Pharmacy and Pharmacology.

[33] Lu M, Han Z, Xu Y, Yao L (2013). In vitro and in vivo anti-tobacco mosaic virus activities of essential oils and individual compounds. Journal of Microbiology and Biotechnology.

[34] Peña A, Rojas L, Aparicio R, Alarcón L, Baptista JG (2012). Chemical composition and antibacterial activity of the essential oil of *Espeletia nana*. Natural Product Communications.

[35] Ko MJ, Nam HH, Chung MS (2019). Conversion of 6-gingerol to 6-shogaol in ginger (*Zingiber officinale*) pulp and peel during subcritical water extraction. Food Chemistry.

[36] Naziruddin MA, Jawaid M, Elias R, Sanny M, Fouad H (2023). Supercritical fluid extraction of torch ginger: Encapsulation, metabolite profiling, and antioxidant activity. Journal of King Saud University–Science.

[37] Semwal RB, Semwal DK, Combrinck S, Viljoen AM (2015). Gingerols and shogaols: Important nutraceutical principles from ginger. Phytochemistry.

[38] Austin PJ, Wu A, Moalem TG (2012). Chronic constriction of the sciatic nerve and pain hypersensitivity testing in rats. Journal of Visualized Experiments.

[39] Cheng KI, Chen SL, Hsu JH, Cheng YC, Chang YC (2021). Loganin prevents CXCL12/CXCR4-regulated neuropathic pain via the NLRP3 inflammasome axis in nerve-injured rats. Phytomedicine.

[40] Cioato SG, Medeiros LF, Marques Filho PR, Vercelino R, de Souza A (2016). Long-lasting effect of transcranial direct current stimulation in the reversal of hyperalgesia and cytokine alterations induced by the neuropathic pain model. Brain Stimulation.

[41] Chen JY, Chu LW, Cheng KI, Hsieh SL, Juan YS (2018). Valproate reduces neuroinflammation and neuronal death in a rat chronic constriction injury model. Scientific Reports.

[42] Wen ZH, Huang SY, Kuo HM, Chen CT, Chen NF (2021). Fumagillin attenuates spinal angiogenesis, neuroinflammation, and pain in neuropathic rats after chronic constriction injury. Biomedicines.

[43] Grégoire S, Michaud V, Chapuy E, Eschalier A, Ardid D (2012). Study of emotional and cognitive impairments in mononeuropathic rats: Effect of duloxetine and gabapentin. Pain.

[44] Haranishi Y, Hara K, Terada T (2022). Analgesic potency of intrathecally administered punicalagin in rat neuropathic and inflammatory pain models. Journal of Natural Medicines.

[45] Ji RR, Xu ZZ, Gao YJ (2014). Emerging targets in neuroinflammation-driven chronic pain. Nature Reviews Drug Discovery.

[46] Zelenka M, Schäfers M, Sommer C (2005). Intraneural injection of interleukin-1beta and tumor necrosis factor-alpha into rat sciatic nerve at physiological doses induces signs of neuropathic pain. Pain.

[47] Schäfers M, Sommer C (2007). Anticytokine therapy in neuropathic pain management. Expert Review of Neurotherapeutics.

